# On the spin dependence of detection times and the nonmeasurability of arrival times

**DOI:** 10.1038/s41598-024-53777-8

**Published:** 2024-02-15

**Authors:** Sheldon Goldstein, Roderich Tumulka, Nino Zanghì

**Affiliations:** 1 Departments of Mathematics and Physics, Rutgers University, Hill Center, 110 Frelinghuysen Road, Piscataway, NJ 08854-8019 USA; 2 Fachbereich Mathematik, Eberhard-Karls-Universität Tübingen, Auf der Morgenstelle 10, 72076 Tübingen, Germany; 3 Dipartimento di Fisica, Università di Genova, Via Dodecaneso 33, 16146 Genoa, Italy; 4 Istituto Nazionale di Fisica Nucleare (Sezione di Genova), Genoa, Italy

**Keywords:** Matter waves and particle beams, Quantum mechanics, Theoretical physics

## Abstract

According to a well-known principle of quantum physics, the statistics of the outcomes of any quantum experiment are governed by a Positive-Operator-Valued Measure (POVM). In particular, for experiments designed to measure a specific physical quantity, like the time of a particle’s first arrival at a surface, this principle establishes that if the probability distribution of that quantity does not arise from a POVM, no such experiment exists. Such is the case with the arrival time distributions proposed by Das and Dürr, due to the nature of their spin dependence.

## Introduction

A few years ago, Das and Dürr, in their work^1^, computed probability distributions for the times of first arrival of a spin-1/2 particle, moving in an axially symmetric region according to an axially symmetric dynamics, at an axially symmetric surface in that region. The calculation was conducted within the framework of Bohmian mechanics, which provides a well-defined concept of arrival time, in contrast to standard quantum mechanics.

The probability distributions they found depend upon the spin of the particle and for some choices of the spin the distribution exhibits intriguing characteristics. They assumed that the arrival times could be measured using appropriate detectors. In fact, it was partly on the basis of the nature of the spin dependence that they argued for this assumption.

However, on the basis of the spin dependence, we prove here that their assumption regarding the measurability of arrival times is false: there can exist no such detectors. This conclusion is grounded in a well-known principle: The statistics of the outcomes of any quantum experiment are governed by a Positive-Operator-Valued Measure (POVM). This principle is considered a fundamental tenet in standard quantum mechanics, as exemplified in references such as^2^. Moreover, it is a theorem within the framework of Bohmian mechanics^3^.

In the specific context of an experiment designed to measure a particular physical quantity, like the particle’s time of first arrival at a surface, this principle establishes that if the probability distribution of that quantity does not arise from a POVM, no such experiment exists. As we show, such is the case with the arrival time distributions found in^1^, due to the nature of their spin dependence.

## Methods

### Necessary condition on spin dependence

Consider an experimental setup involving a spin-1/2 particle. The outcome statistics is given by a POVM $\mathcal {O}= \mathcal {O}(dt)$, i.e., when the quantum state of the particle is $\Psi$, the distribution of results can be expressed as $\langle \Psi | \mathcal {O} | \Psi \rangle$.

Now, let us introduce $\textbf{n}$, an arbitrary unit vector in three-dimensional space. A spinor $|\textbf{n}\rangle$ in $\mathbb {C}^2$ is defined uniquely up to a phase by the condition $\textbf{n} = \langle \textbf{n} | \varvec{\sigma } |\textbf{n}\rangle$, where $\varvec{\sigma }=(\sigma _x, \sigma _y, \sigma _z)$ constitutes the vector of Pauli matrices.

Suppose that $\Psi$ takes the form of a product state,

1 $$\begin{aligned} \Psi = \psi \otimes |\textbf{n}\rangle , \end{aligned}$$

where $\psi$ accounts for the translational degrees of freedom. When considering $\psi$ as fixed and the spin state $|\textbf{n}\rangle$ as variable, the spin-dependent aspect of the result’s statistics can be encapsulated by the *spin POVM* $O=O(dt) =\langle \psi |\mathcal {O}(dt)|\psi \rangle$. Consequently, the statistics of the result for the state defined in Eq. (1) can be expressed as

2 $$\begin{aligned} P_\textbf{n} = P_\textbf{n}(dt) = \langle \textbf{n} | O (dt) |\textbf{n}\rangle . \end{aligned}$$

This severely constrains the possible spin dependence of the outcome statistics $P_\textbf{n}$. Since $|\textbf{n}\rangle$ and $|\mathbf{-n}\rangle$ are orthogonal, we have that

3 $$\begin{aligned} P_\textbf{n} + P_\mathbf{-n}=\textrm{Tr}(O), \end{aligned}$$

so that it does not depend on the chosen direction $\textbf{n}$ of the spin vector. We thus arrive at the following necessary condition:

4 $$\begin{aligned}{} & {} { Let }\, \mathscr {P}_\textbf{n}=\mathscr {P}_\textbf{n} (dt)\, be\, any\, spin\text{-}dependent\, distribution,\, for\, example\, that\, of\, a\, physical\, quantity\,\nonumber \\{} & {} \quad such\, as\, an\, arrival\, time\, for\, a\, system\, in\, the\, state\, (1).\, For\, this\, to\, arise\, from\, a\, spin\, POVM\,\nonumber \\{} & {} \quad it\, must\, be\, the\, case\, that\, \mathscr {P}_\textbf{n} + \mathscr {P}_\mathbf{-n}\, { does\, not\, depend\, on\, the\, chosen\, direction\, } \textbf{n}\, { of\, the\, spin\, vector. } \end{aligned}$$

### Axial symmetry

Assuming axial symmetry, let us consider a unit vector $\textbf{a}$ aligned with the symmetry axis and another unit vector $\textbf{b}$ perpendicular to $\textbf{a}$. We write $P_{\uparrow }$ for $P_\textbf{a}$, $P_{\downarrow }$ for $P_{-\textbf{a}}$, and $P_{\rightarrow }$ for $P_\textbf{b}$, with similar notation for $\mathscr {P}$. Axial symmetry implies that $\mathscr {P}_\textbf{b}=\mathscr {P}_{-\textbf{b}}$. Thus, choosing $\textbf{a}$ and $\textbf{b}$ for $\textbf{n}$ in (4), we have that

5 $$\begin{aligned}{} & {} {Under\, axial\, symmetry,\, the\, spin\text{-}dependent\, distribution\, } \mathscr {P}_\textbf{n}\, {of\, a\, physical\, quantity\, in\, the\, state\, } (1)\nonumber \\{} & {} \quad {can\, arise\, from\, a\, spin\, POVM\, only\, if\, } \end{aligned}$$

$$\begin{aligned} \mathscr {P}_{\uparrow } + \mathscr {P}_{\downarrow } = 2\mathscr {P}_\rightarrow . \end{aligned}$$

Suppose now that, in addition to axial symmetry, we have also that $\mathscr {P}_{\uparrow } = \mathscr {P}_{\downarrow }$. In this case (5) becomes the following:

6 $$\begin{aligned} {Under\, axial\, symmetry,\, if\, } \mathscr {P}_{\uparrow } = \mathscr {P}_{\downarrow },\, { then\, } \mathscr {P}_\textbf{n}\, { can\, arise\, from\, a\, spin\, POVM\, only\, if\, } \mathscr {P}_{\uparrow }= \mathscr {P}_{\rightarrow }. \end{aligned}$$

## Conclusion

### Arrival times in Bohmian mechanics

 In the context of Bohmian mechanics, arrival times are well-defined, and the crucial question revolves around their measurability. In^1^, Das and Dürr calculated the arrival time distributions $\mathscr {P}_\textbf{n}$ and found intriguing spin-dependent characteristics. In addition to having axial symmetry, the arrival time distributions were such that $\mathscr {P}_{\uparrow }= \mathscr {P}_{\downarrow }$. Moreover, they found that $\mathscr {P}_\uparrow \ne \mathscr {P}_\rightarrow$ (in fact, dramatically so). Thus, by (6), the arrival times distributions don’t arise from a POVM, and hence the arrival times are not measurable.

### Approximate measurement

 If a physical quantity can’t be measured with arbitrary precision, it still might be possible to perform an approximate measurement of the quantity. It is natural to then ask, given $\mathscr {P}_\textbf{n}$, how closely this can be approximated by a spin POVM. We have no definitive answer to this question, but we can provide a simple lower bound on the size of the error.

Let

7 $$\begin{aligned} \Delta = \sup _{\textbf{n},\textbf{m}}\Vert (\mathscr {P}_\textbf{n} + \mathscr {P}_\mathbf{-n})-(\mathscr {P}_\textbf{m} + \mathscr {P}_\mathbf{-m})\Vert , \end{aligned}$$

where $\Vert \cdot \Vert$ denotes, say, the total variation norm—$L^1$ for absolutely continuous measures. Then it is easy to see that any approximating spin POVM must yield an error of at least $\Delta /4$ (or $\Delta /4-\epsilon$ if the continuity of $\mathscr {P}_\textbf{n}$ as a function of $\textbf{n}$ is not assumed), in the sense that for any (approximating) spin POVM *O*, there must be a direction $\textbf{n}$ such that $\Vert \mathscr {P}_\textbf{n}- \langle \textbf{n} | O |\textbf{n}\rangle \Vert \ge \Delta /4-\epsilon$. (Indeed, by (7) there are $\textbf{n}$ and $\textbf{m}$ such that the norm in (7) is within $4\epsilon$ of the supremum $\Delta$. By (3), replacing $\mathscr {P}_\textbf{n}$ in (7) by $\mathscr {P}_\textbf{n}-\langle \textbf{n}|O|\textbf{n}\rangle$, and likewise for $-\textbf{n},\textbf{m},-\textbf{m}$, leaves the norm in (7) unchanged. By the triangle inequality, this norm is $\le$ the sum of $\Vert \mathscr {P}_\textbf{n}-\langle \textbf{n}|O|\textbf{n}\rangle \Vert$ and the corresponding terms for $-\textbf{n},\textbf{m},-\textbf{m}$; thus, not all of the 4 summands can be $< \Delta /4-\epsilon$.)

Thus for any POVM *O*, with associated probability distributions $P_\textbf{n}$, we have that

8 $$\begin{aligned} \Vert \mathscr {P}-P\Vert \equiv \sup _\textbf{n} \Vert \mathscr {P}_\textbf{n}- P_\textbf{n}\Vert \ge \Delta /4. \end{aligned}$$

As a consequence, for the model of^1^, in which $\mathscr {P}_{\uparrow } = \mathscr {P}_{\downarrow }$, any potential arrival time *measurement* must involve an error of at least $\Vert \mathscr {P}_{\rightarrow }-\mathscr {P}_{\uparrow }\Vert /2$ in the above sense. Since this quantity is not negligible, the family of the $\mathscr {P}_\textbf{n}$ can’t be close to any family of $P_\textbf{n}$, contrary to the expectation of Das and Dürr that the presence of detectors would cause only a mild disturbance^1^.

### Inversion symmetry

In addition to obeying $\mathscr {P}_{\uparrow }= \mathscr {P}_{\downarrow }$, the arrival time distributions $\mathscr {P}_\textbf{n}$ found in^1^ have full inversion symmetry, obeying $\mathscr {P}_\textbf{n}=\mathscr {P}_\mathbf{-n}$ for all $\textbf{n}$. It follows from (4) that under this *axial-inversion* symmetry, the arrival time distributions, if measurable, could involve no spin dependence whatsoever.

### Chiral symmetry

In fact, a more detailed examination of the form of an axially symmetric spin POVM shows that, assuming only axial symmetry, $P_\textbf{n}$ can have no spin dependence at all if it obeys $P_{\uparrow }=P_{\downarrow }$, an expression of chiral symmetry, i.e., symmetry between the clockwise and the counter-clockwise sense of rotation about the $\textbf{a}$-axis. (Indeed, any axially symmetric POVM *O* must be of the form $O=AI+B\textbf{a}\cdot \varvec{\sigma }$, so that $O=P_\rightarrow I + \frac{1}{2}(P_\uparrow -P_\downarrow ) \textbf{a}\cdot \varvec{\sigma }$.)

Thus any detector that respects the axial and chiral symmetry of the model in^1^, regardless of how accurate or inaccurate it may be, will find no spin dependence for *detection* time statistics. In particular such detectors will reveal no difference in what they find for directions in which the *arrival* time distributions have intriguing characteristics and those in which they don’t.

## Data Availability

All data generated or analysed during this study are included in this published article.
